# Functional Cooperation between Vitamin D Receptor and Runx2 in Vitamin D-Induced Vascular Calcification

**DOI:** 10.1371/journal.pone.0083584

**Published:** 2013-12-12

**Authors:** Min-Su Han, Xiangguo Che, Gyoung-ho Cho, Hye-Ri Park, Kyung-Eun Lim, Na-Rae Park, Jung-Sook Jin, Youn-Kwan Jung, Jae-Hwan Jeong, In-Kyu Lee, Shigeaki Kato, Je-Yong Choi

**Affiliations:** 1 Department of Biochemistry and Cell Biology, WCU and BK21 plus programs, CMRC, Skeletal Diseases Genome Research Center, School of Medicine, Kyungpook National University, Daegu, Republic of Korea; 2 Department of Internal Medicine, Division of Endocrinology, School of Medicine, Kyungpook National University, Daegu, Republic of Korea; 3 Institute of Molecular and Cellular Biosciences, University of Tokyo, Tokyo, Japan; Nihon University School of Medicine, Japan

## Abstract

The transdifferentiation of vascular smooth muscle cells (VSMCs) into osteoblast-like cells has been implicated in the context of vascular calcification. We investigated the roles of vitamin D receptor (Vdr) and *runt-related transcription* factor *2* (*Runx2*) in the osteoblastic differentiation of VSMCs in response to vitamin D_3_ using *in vitro* VSMCs cultures and *in vivo* in *Vdr* knockout (*Vdr* ^*-/-*^) and *Runx2 carboxy-terminus* truncated heterozygous (*Runx2* ^*+/ΔC*^) mice. Treatment of VSMCs with active vitamin D_3_ promoted matrix mineral deposition, and increased the expressions of Vdr, Runx2, and of osteoblastic genes but decreased the expression of smooth muscle myosin heavy chain in primary VSMCs cultures. Immunoprecipitation experiments suggested an interaction between Vdr and Runx2. Furthermore, silencing *Vdr* or *Runx2* attenuated the procalcific effects of vitamin D_3_. Functional cooperation between Vdr and Runx2 in vascular calcification was also confirmed in *in vivo* mouse models. Vascular calcification induced by high-dose vitamin D_3_ was completely inhibited in *Vdr* ^*-/-*^ or *Runx2* ^*+/ΔC*^ mice, despite elevated levels of serum calcium or alkaline phosphatase. Collectively, these findings suggest that functional cooperation between Vdr and Runx2 is necessary for vascular calcification in response to vitamin D_3_.

## Introduction

Vascular calcification (VC) is a prevalent and progressive pathological process. VC is caused by a failure to maintain homeostasis of the vasculature in diseases like atherosclerosis, diabetes, chronic kidney disease (CKD), and during normal aging [1-3]. Inflammation, reactive oxygen species, and hyperlipidemia are major contributors to the pathogenesis of VC [4-7]. Interestingly, a number of studies have shown VC recapitulates embryonic osteogenesis, and that vascular smooth muscle cells (VSMCs) are able to transdifferentiate into both osteogenic and chondrogenic cells [1,8,9]. Furthermore, transdifferentiation, also called lineage reprogramming, has been reported to be responsible for the presence of osteoblast-like cells instead of VSMCs during VC [10]. In addition, the transdifferentiation of VSMCs into osteochondroprogenitor-like cells occurs before mineral deposition and is associated with the onset of osteogenic gene expression [10]. 

As in bone-derived osteoblasts [11-14], *runt-related transcription* factor *2* (*Runx2*) is a regulator of VC-related genes in VSMCs. The expression of *Runx2* and other bone related factors, such as, *osterix* (*Osx*), *alkaline phosphatase (Alp*)*, bone sialoprotein*, *msh homeobox* homolog *2, type I collagen*, and *SRY-box containing gene 9* are elevated in calcified vessels [15-18]. Mouse-based genetic approaches have shown many genes, such as, matrix gla protein (Mgp)*, osteoprotegerin* (*Opg*)*, apolipoprotein E, fetuin, klotho, fibroblast growth* factor *23, SMAD family* member *6*, and *adiponectin* are important regulators of VC [19-22]. On the other hand, the protein levels of smooth muscle cell markers, such as, *smooth muscle* protein *22 alpha*, *smooth muscle alpha actin* (*Sm-α-actin*), and *smooth muscle myosin heavy chain (Smmhc*) are diminished [17]. 

Vitamin D receptor (Vdr) is a candidate VC-related gene because it is involved in calcium and phosphate homeostasis and normal bone metabolism [23,24]. Vdr also regulates the expressions of bone modulating factors, such as, *Runx2*, *receptor activator of NF-kappa-B ligand* (*Rankl*)*, osteocalcin* (*Ocn*)*, and osteopontin* (*Opn*) [25-27]. In *transcriptional intermediary* factor *1 alpha* null mice, VC was induced by upregulating the expressions of *Vdr* target genes [28], and clinically, VC can be induced by vitamin D deficiency or excess [29,30]. For example, CKD patients show a ‘U-shaped’ VC development tendency with respect to serum vitamin D levels, which suggests that correct vitamin D dosage is important [31]. Accordingly, the dietary intake allowance of vitamin D should be strictly controlled CKD patients [32]. 

Although the above-mentioned studies suggest the importance of *Vdr* and *Runx2* in the osteoblastic differentiation of VSMCs, it is not clear whether both are necessary. In the present study, we examined their contributions to VC using *Vdr* ^*-/-*^ and *Runx2 carboxy-terminus* deletion mutant (*Runx2* ^*+/ΔC*^) mice [12,33]. 

## Methods

### Materials

Cholecalciferol (vitamin D_3_), calcitriol (1,25(OH)_2_D_3_), β-glycerophosphate, ascorbic acid, and Alp staining kits were purchased from Sigma Aldrich (St. Louis, MO). Calcium, phosphate, and Alp assay kits were obtained from Bio Assay Systems (Hayward, CA, USA). Nuclear and cytoplasmic extraction reagents were purchased from Thermo Scientific (Rockford, IL, USA). Immunoblotting detection kits were purchased from GE Health Care (Bucks, UK). Mouse monoclonal antibodies for Vdr (Santa Cruz, CA, USA), and Runx2, rabbit polyclonal antibodies for Runx2 (Santa Cruz), Ocn (Santa Cruz), Mgp (Santa Cruz), Smmhc (Abcam), goat polyclonal antibodies for Lamin B1 (Santa Cruz), goat anti- mouse, anti-rabbit IgG and donkey anti-goat IgG conjugated with horseradish peroxidase (HRP; Santa Cruz), and mouse and rabbit normal IgG (Vector Laboratories, Burlingame, CA, USA) were purchased as indicated. Iso-IHC DAB Kits (InnoGenex, San Ramon, CA, USA) were used for the immunohistochemical analyses of mouse tissues using mouse primary antibodies. CYBR green PCR master mix was obtained from Takara Co. (Shiga, Japan). 

### In vitro cell culture and osteogenic differentiation

Primary VSMCs isolated from mouse or rat aortas were obtained using a previously described explant culture method [34]. Briefly, 8-week-old mouse or rat aortas were extracted and cut into small pieces (1-2 mm^3^). These were then incubated in 0.1% collagenase II (Worthington, NJ, USA) for 5 minutes at 37°C and placed in 100 mm culture dishes and cultured in Dulbecco’s Modified Eagle’s medium (DMEM, Invitrogen, Carlsbad, CA) supplemented with 4.5 g/l glucose, 10 mmol/l sodium pyruvate, 100 U/ml penicillin and 100 μg/ml streptomycin, and 50% fetal bovine serum (FBS, Invitrogen) at 37°C in a 5% CO_2_/95% air atmosphere. Cells were maintained in DMEM containing 4.5 g/l glucose, 10 mmol/l sodium pyruvate, 100 U/ml penicillin, 100 μg/ml streptomycin, and 10% FBS (maintaining medium). To treat VSMCs with vitamin D_3_ or 1,25(OH)_2_D_3_, cells were seeded (2 x 10^4^ cells/well) on 6-well culture plates and cultured overnight in maintaining medium. They were then cultured in phenol red free DMEM containing 4.5 g/l glucose, 10 mmol/l sodium pyruvate, 100 U/ml penicillin, 100 μg/ml streptomycin, and 10% charcoal-dextran stripped FBS and treated with vitamin D_3_ or 1,25(OH)_2_D_3_ for 24 hours. 

For the calcification experiment, VSMCs were seeded (5 x 10^4^ cells/well) on 6-well culture plates and cultured in maintaining medium containing 10 mmol/l β-glycerophosphate and 50 μg/ml ascorbic acid (osteogenic medium) with or without 1,25(OH)_2_D_3_ (10^-7^ mol/l) for 2-4 weeks. Of the concentrations of 1,25(OH)_2_D_3_ examined (0, 10^-10^, 10^-9^, 10^-8^, or 10^-7^ mol/l), we chose 10^-7^ mol/l 1,25(OH)_2_D_3_ because it induced a prominent increase in the expressions of Vdr and Runx2 in preliminary experiments.

### Alp and von Kossa staining of VSMCs

To detect mineralization, VSMCs were cultured in osteogenic medium for 14 days, washed with PBS, and fixed 2% formaldehyde (Junsei Chemical Co) at room temperature (RT) for 10 minutes. After washing with PBS, cells were treated with Alp staining solution and incubated at 37°C for 30 minutes. Cells were then cultured in osteogenic medium for 21 days and von Kossa stained, as previously described [35]. Briefly, cells were fixed with 0.1% glutaraldehyde (Sigma Aldrich) for 15 minutes at RT, washed with autoclaved/deionized Milli Q water twice, incubated with 5% silver nitrate (Junsei Chemical Co, Tokyo) under an UV lamp (UVP, CA, USA) for 30 minutes at RT, washed twice with water, treated with 2.5% sodium thiosulfate (Sigma Aldrich) to remove nonspecific signals, and processed for scanning (Epson Perfection V10 scanner; Seico Epson Corp, Nagano, Japan). 

### Real-time RT-PCR analysis

Real-time RT-PCR was used to quantify the relative mRNA levels of various genes, such as, *Gapdh, Ocn, Opg, Osx, Rankl, Runx2, Smmhc*, and *Vdr* using a Roche LightCycler 480 (Indianapolis, IN, USA). Primers were designed using ABI Primer Express version 2.0 (Carlsbad, CA, USA). Briefly, total RNA (1-5 μg) was extracted from aortas and reverse transcribed cDNA was used as a template in each PCR. CYBR green PCR master mix (4 μl, Takara Co) and specific primers were combined with templates, and a negative control (no template) was included in each assay. PCR was performed over 50 cycles of 10 minutes at 95°C, 15 seconds at 95°C, 15 seconds at 60°C, and 15 seconds at 72°C. The primers used for real-time RT-PCR are described in Table 1.

**Table 1:** Listing of the real-time RT-PCR primers used to quantify the relative expressions of bone related genes in the aortas of vitamin D_3_-induced VC mice.

**Genes**	**NCBI Gene Accession number**	**Primer pair (forward/reverse)**	**Product size (bp)**	**Annealing temperature (℃)**
*Gapdh*	NM_008084	GCATCTCCCTCACAATTTCCA	101	60
	.2	GTGCAGCGAACTTTATTGATGG		
*Ocn*	NM_001037	TTCTGCTCACTCTGCTGACCCT	102	60
	939.1	CCTGCTTGGACATGAAGGCTT		
*Opg*	NM_008764	CACAAGAGCAAACCTTCCAGC	104	60
	.3	GCTGCTTTCACAGAGGTCAATG		
*Osx*	NM_130458	AGAGGTTCACTCGCTCTGACGA	115	60
	.3	TTGCTCAAGTGGTCGCTTCTG		
*Rankl*	NM_011613	GATTTTTCAAGCTCCGAGCTG	108	60
	.3	CCTGAACTTTGAAAGCCCCAA		
*Runx2*	NM_001146	AATTGCAGGCTTCGTGGTTG	136	60
	038.2	TCCCCTGAATGGCTGTATGGT		
*Smmhc*	NM_013607	GCGCAATACCACGCCTAACTT	128	60
	.2	AGATGCGGATGCCTTCCAA		
*Vdr*	NM_009504	AGCAACAGCACATTATCGCCAT	104	60
	.4	TACGTCTGCACGAATTGGAGG		

Primers were designed using ABI Primer Express version 2.0 (Carlsbad).

### Immunoblotting analysis

Whole cell lysates were prepared using RIPA buffer [150 mmol/l NaCl, 1% Triton X-100, 1% sodium deoxycholate, 0.1% SDS, 50 mmol/l Tris-HCl (pH7.5), and 2 mmol/l EDTA (pH 8.0)]. Cytosolic and nuclear extracts (NEs) were prepared using NE-PER nuclear and cytoplasmic extraction kits (Thermo Scientific). Immunoblottings were carried out as previously described [36]. The following antibodies were used: Vdr (diluted 1:2,000, Santa Cruz), Runx2 (diluted 1:1,000, Santa Cruz), Smmhc (diluted 1:1,000, Abcam Inc, Cambridge, MA, USA), Sm-α-actin (diluted 1:3,000, Abcam Inc), β-actin (diluted 1:3,000, Santa Cruz), and Lamin B1 (diluted 1:2,000, Santa Cruz). 

### Adenovirus expressing Runx2

To overexpress Runx2, we used an adenovirus expressing Runx2, as previously described [37]. Briefly, 70% confluent primary cultured VSMCs from a mouse or rat were plated in 100 mm plates and infected with adenovirus expressing Runx2 (50 moi) in maintaining medium without FBS for 4 hours. Cells were then cultured for 42 hours in experimental medium with/without 1,25(OH)_2_D_3_.

### RNA interference analysis

To knock-down *Vdr* or *Runx2* mRNA, we used the ON-TARGET plus small interfering RNA (siRNA) system (Dharmacon, Chicago, IL). Briefly, 50% confluent primary cultured VSMCs were seeded in 6-well plates, cultured overnight, and transfected for 24 hours (for mRNA analysis) with siRNAs (25 or 50 nmol/l) for *Vdr* or *Runx2* mRNAs with/without 1,25(OH)_2_D_3_.

### Co-immunoprecipitation analysis

HEK293 cells (70% confluent) were seeded in 100 mm plates, cultured overnight, and transiently transfected with pCS4-3Flag-Runx2 or empty vector. After 4~6 hours of transfection, cells were cultured in the presence or absence of 10^-7^ mol/l 1,25(OH)_2_D_3_ for 24 hours and NEs were prepared using the NE-PER kit. Co-immunoprecipitation analysis was performed as previously described [38]. To remove non-specific binding proteins, protein G Sepharose (Santa Cruz) slurry was added to 130 μg aliquots of NEs and incubated at 4°C for 2 hours with rotation. After a spin down, mouse monoclonal anti-Vdr antibody or mouse normal IgG was added to supernatants at 4°C for 1 hour. Protein G Sepharose beads were then added to mixed supernatants and incubated at 4°C overnight with rotation. The beads were then rinsed five times with wash buffer [10 mmol/l Tris (pH 7.8), 150 mmol/l NaCl, 1 mmol/l EDTA, 1 mmol/l EGTA (pH 8.0), 5 mmol/l NaF, 1 mmol/l Na_3_VO_4_, 1 mmol/l Na_4_P_2_O_7_, 1% Triton X-100, 0.5% NP-40, and protease inhibitor cocktail (Calbiochem, Darmstadt, Germany)]. 6X SDS-sample loading buffer (30 μl) was added to the washed protein G beads which were then heated at 100°C for 5 minutes. Co-immunoprecipitated 3Flag-Runx2 was detected by Western blotting using an anti-Flag monoclonal antibody (Sigma Aldrich).

### Animals and ethics statement

C57BL/6N mice were supplied by KOATECH (GyeongGi-Do, Korea). Vdr^-/-^ mice were fed with a high calcium rescue diet as previously described [33,39]. *Runx2* ^*+/ΔC*^ mice were fed a normal diet [37]. Animals were maintained under a 12-hour light:12-hour darkness cycle at 22~25°C in specific pathogen-free (SPF) conditions and fed standard rodent chow and water *ad libitum*. All animal experiments were approved by Kyungpook National University (KNU) and all procedures related to animal experimentation were carried out in strict accordance with the guidelines issued by the Institutional Animal Care and Use Committee of KNU (No. KNU-2011-67). Surgery was performed under avertin or isoflurane anesthesia, and all efforts were made to minimize suffering.

### Vitamin D-mediated VC mouse model

This model is commonly used to investigate VC because medial calcification is easily produced [40]. Normal C57BL/6N, *Vdr* ^*-/-*^, or *Runx2* ^*+/ΔC*^ [12] mice were injected subcutaneously with 6 or 8 x 10^5^ IU/kg of vitamin D_3_ daily for three days (n=5-8 per group). The vitamin D_3_ solution (2.64 x 10^6^ IU) was prepared as follows. Vitamin D_3_ (66 mg) in 200 μl of absolute ethanol was mixed with 1.4 ml of cremophor (Sigma Aldrich) at RT, and then with 18.4 ml of sterilized water containing 750 mg of dextrose at RT. After injecting vitamin D_3_, body weights were checked daily until sacrifice.

### Alizarin red S and von Kossa staining and immunohistochemical analyses

Mice were sacrificed at 9 or 10 days after first vitamin D_3_ injection, and aortas were dissected and stained with 0.005% Alizarin red S in 0.5% KOH for 24 hours [41]. For histological analysis, mice were sacrificed at day 9 or 10 after first vitamin D_3_ injection, and aortas were fixed in 4% formaldehyde overnight. For paraffin sections, fixed aortas were dehydrated and embedded in paraffin and cut into 3 μm sections. von Kossa staining was performed to detect mineralization as described previously [42]. Deparaffinized sections were washed in Milli-Q water for 30 minutes, incubated in 1% aqueous silver nitrate solution under a UV lamp for 30 minutes, and washed with water three times at RT. Non-specific staining was removed by then treating sections with 2.5% sodium thiosulfate for 5 minutes at RT. Immunohistochemical analyses were carried out as previously described [43] or by following the manufacturer’s instructions (InnoGenex) as follows. Briefly, endogenous peroxidase activity was quenched in antigen-retrieved sections using 3% H_2_O_2_. After blocking with 1% BSA for 1 hour at RT, sections were incubated with the indicated primary antibodies (Vdr, Runx2, Runx2, Mgp, and Smmhc) diluted at 1:200~1:1000 for 16 hours at 4°C and then with goat anti-rabbit IgG conjugated with HRP (DakoCytomation, Glostrup, Denmark) for 1 hour at RT. To visualize signals, sections were developed using a DAB substrate-chromogen system (DakoCytomation). Rabbit and mouse normal IgG were used as controls at the same dilution as the appropriate primary antibodies. Microscopy was conducted using a light microscope and the AxioVison program (Carl-Zeiss, Jena, Germany).

### Serum biochemical analysis

Calcium, phosphate, and Alp levels in mouse sera were analyzed using commercially available kits (Bio Assay Systems). For calcium analysis, 5 μl of serum samples or of standards were transferred into the wells of a clear bottom 96-well-plate (Corning Inc, Corning, NY, USA). Working reagent (200 μl) was added, incubated for 3 minutes at RT, and absorbances were then measured at 612 nm using a microplate reader (Tecan, Austria, GmbH, Austria). For phosphate analysis, 2 μl serum samples diluted with 48 μl of autoclaved/deionized Milli-Q water or 50 μl of water (blank) were transferred to the wells of a clear bottom 96-well plate. Reagents (100 μl) were added, incubated for 30 minutes at RT, and absorbances were measured at 620 nm using a microplate reader. For Alp analysis, 10 μl serum samples were transferred to the wells of a clear bottom 96-well plate. Working solution (190 μl) was added and absorbances were measured immediately at 405 nm (time = 0) and again 4 minutes (time = 4 minutes) later using a microplate reader.

### Statistics

Statistical analyses were performed by unpaired Student’s *t*-test using SigmaPlot 10.0. We considered P values <0.05 as statistically significant. 

## Results

### Effects of 1,25(OH)_2_D_3_ on VSMC calcification

**Figure 1:**
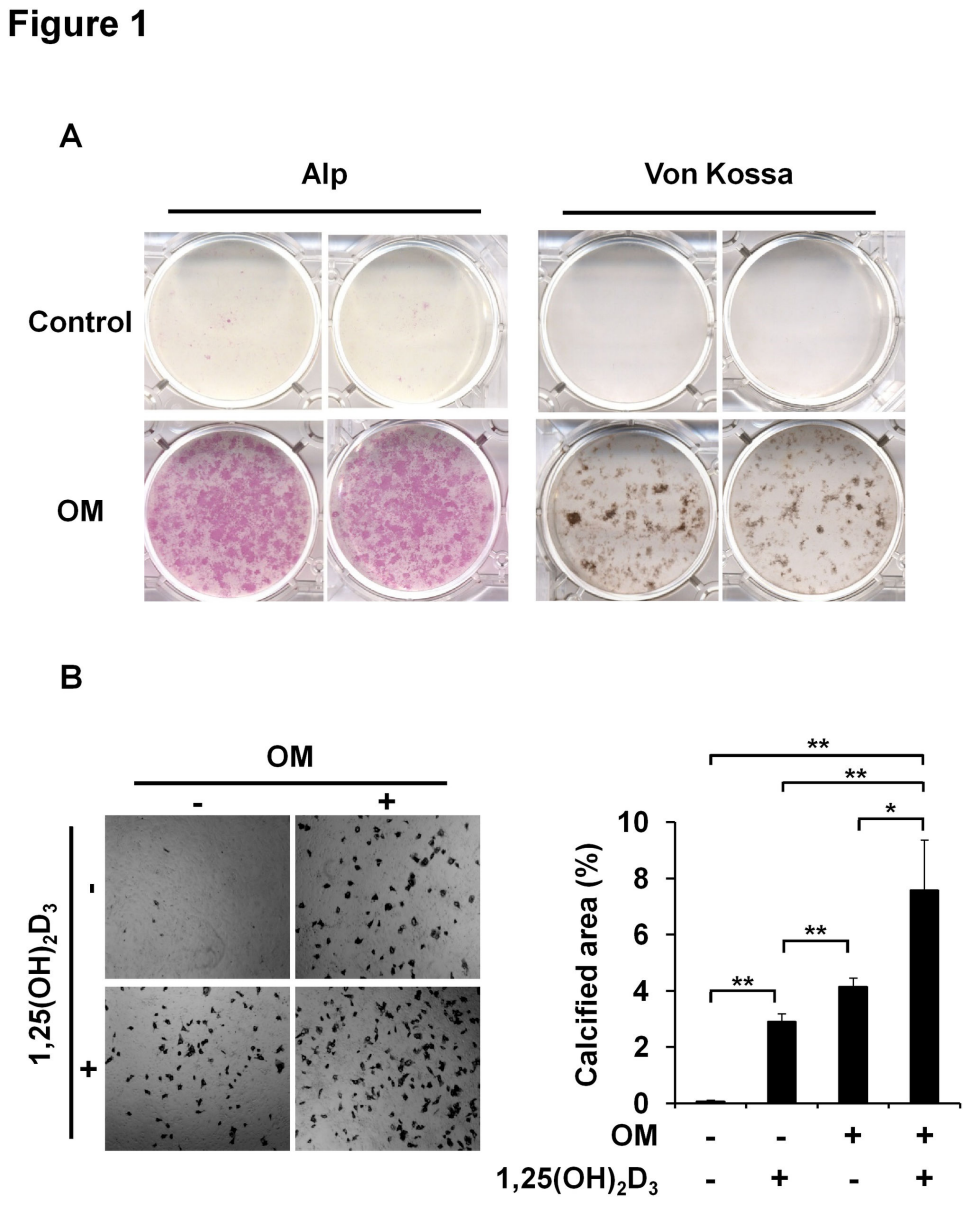
Effects of 1,25(OH)_2_D_3_ on VSMC calcification. (A) Primary mouse VSMCs were cultured in osteogenic medium (OM) or non-osteogenic control medium (Control). Alkaline phosphatase (Alp) and von Kossa staining were performed on culture day 21. (B) VSMCs were cultured with or without 1,25(OH)_2_D_3_ (10^-7^ mol/l) and von Kossa stained on day 21. Results are the means ± SD of three separate experiments (Right panel). Statistical analysis was performed using the unpaired Student’s *t*-test. *P<0.05 or **P<0.01 versus the untreated condition.

To test the osteoblastic differentiation of VSMCs, primary mouse VSMCs were cultured in osteogenic media, and then Alp activity and mineralization were assessed. Osteogenic media promoted Alp activity and bone nodule formation (Figure 1A), and treatment with 1,25(OH)_2_D_3_ further enhanced differentiation of VSMCs into osteoblast-like cells (Figure 1B). It has been reported that endothelial cells can transdifferentiate into osteoblast-like cells [44], and to test this possibility, primary endothelial cells derived from human umbilical cord vein were cultured in osteogenic media treated with 1,25(OH)_2_D_3_. However, osteoblastic differentiation was not observed in endothelial cells (data not shown).

### Effects of 1,25(OH)_2_D_3_ on the protein and mRNA levels of Vdr and Runx2

**Figure 2:**
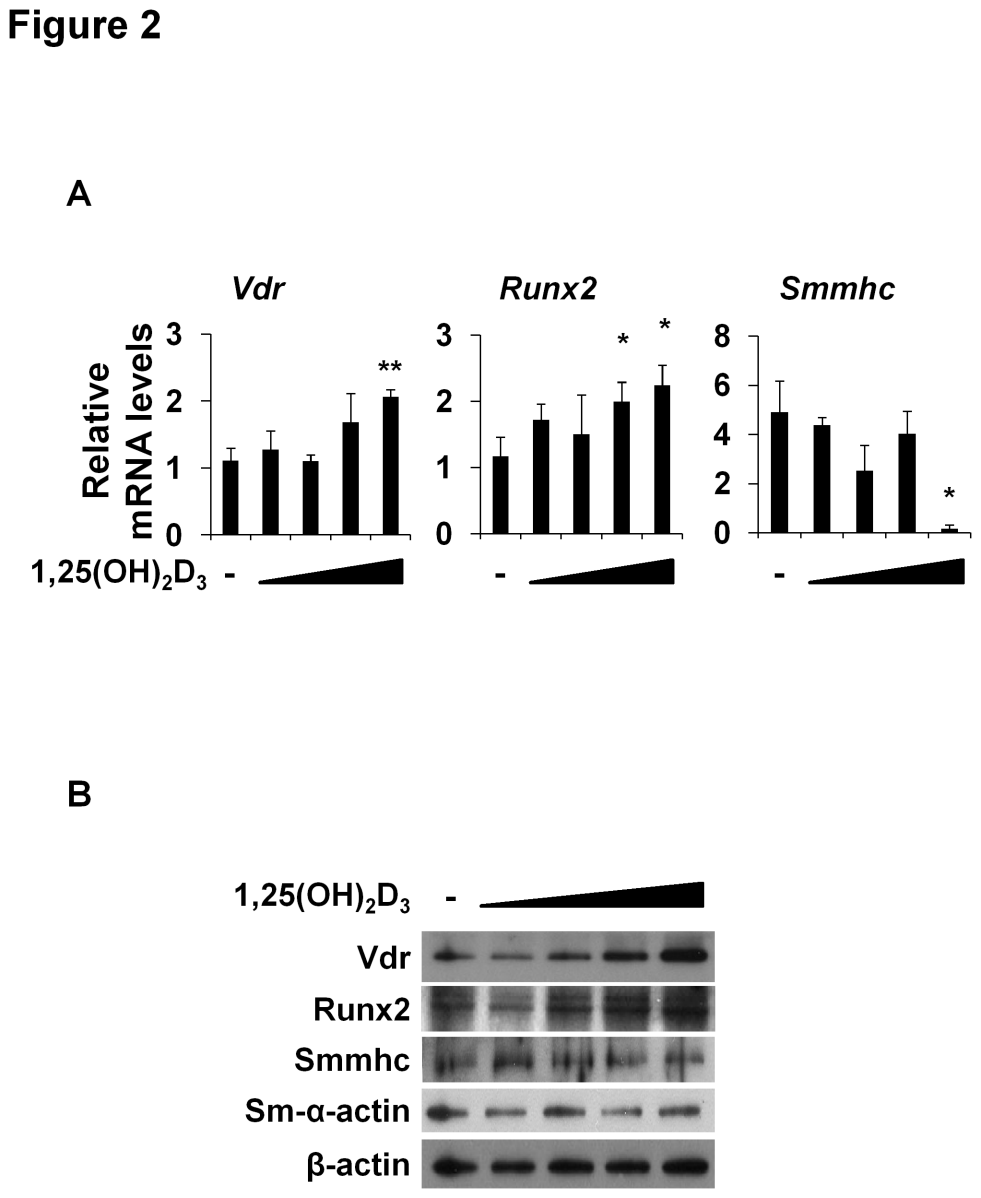
Effects of 1,25(OH)_2_D_3_on the expressions of Vdr and Runx2. (A) Primary cultured mouse VSMCs were treated with 1,25(OH)_2_D_3_ (0, 10^-10^,10^-9^,10^-8^, or 10^-7^ mol/l) for 24 hours. Relative mRNA levels of *Vdr*, Runx2, and Smmhc were measured by real-time RT-PCR. (B) Protein levels of Vdr, Runx2, Smmhc, and Sm-α-actin were measured in total cell lysates by immunoblotting after treatment with 1,25(OH)_2_D_3_for 24 hours. β-Actin was used as an internal control. Statistical analysis was performed using the unpaired Student’s *t*-test. *P<0.05 or **P<0.01 versus the untreated condition.

To determine the molecular mechanism responsible for 1,25(OH)_2_D_3_-mediated osteoblastic differentiation, mouse VSMCs were treated with increasing concentrations (0, 10^-10^, 10^-9^, 10^-8^, or 10^-7^ mol/l) of 1,25(OH)_2_D_3_ (Figure 2). Since Runx2 is an essential player in osteoblastic differentiation [11-14], we assessed levels of Runx2 and Vdr after treating VSMCs with 1,25(OH)_2_D_3_ for 24 hours. The mRNA levels of *Vdr* and *Runx2* were increased by 1,25(OH)_2_D_3_ (Figure 2A), and by vitamin D_3_ at 10^-5^ mol/l (data not shown). Vdr and Runx2 protein levels were also increased by 1,25(OH)_2_D_3_ (Figure 2B). In contrast, 1,25(OH)_2_D_3_ decreased *Smmhc* mRNA levels not its protein levels (Figure 2B). These results indicate that 1,25(OH)_2_D_3_ increases the expressions of Vdr and Runx2 in VSMCs.

### Reciprocal regulation between Vdr and Runx2 by 1,25(OH)_2_D_3_

**Figure 3:**
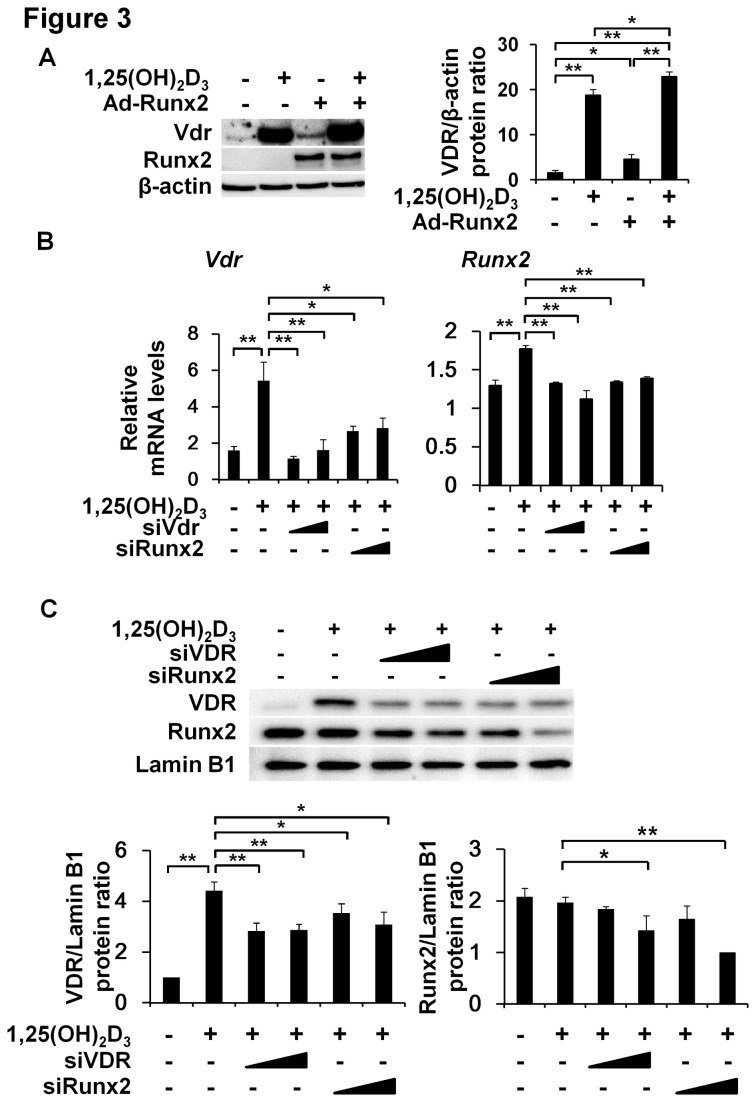
Reciprocal regulation of Vdr and Runx2 by 1,25(OH)_2_D_3_. (A) Rat VSMCs were infected with adenovirus expressing LacZ (Ad-LacZ) or Runx2 (Ad-Runx2) vectors (50 moi) for 4 hours and then treated with or without 1,25(OH)_2_D_3_ (1x10^-7^ mol/l) for 42 hours. The expression levels of Vdr and Runx2 were detected by immunoblotting. (B) Sub-confluent mouse VSMCs were seeded in 6-well plates, cultured overnight, and transfected with siRNAs (25 and 50 nmol/l) for *Vdr* or Runx2 mRNAs and then treated with 1,25(OH)_2_D_3_ (except the control) for 24 hours. Total RNAs were subjected to real-time RT PCR. (C) Mouse VSMCs cultured for 48 hours were analyzed for Vdr and Runx2 protein levels. Lamin B1 was used as internal control. Results are the means ± SD of three independent experiments. Statistical analysis was analyzed using the unpaired Student’s *t*-test. *P<0.05; **P<0.01.

To determine the relationship between Vdr and Runx2, VSMCs derived from rat aortas were infected with adenovirus expressing Runx2 and treated with 1,25(OH)_2_D_3_ for 42 hours. The overexpression of Runx2 was found to increase Vdr expression, and this was further enhanced by treatment with 1,25(OH)_2_D_3_ (Figure 3A). We also performed knockdown experiments to determine whether Runx2 and Vdr have a reciprocal regulatory function. VSMCs derived from mouse aorta were treated with siRNA for *Vdr* or *Runx2* and then their mRNA expressions induced by 1,25(OH)_2_D_3_ were assessed. Knocking down *Vdr* or *Runx2* using siRNAs reduced both of their expressions at the mRNA and protein levels (Figures 3B and 3C), indicating the presence of reciprocal regulation between Vdr and Runx2 in VSMCs.

### Effects of the Vdr/Runx2 interaction on the expressions of osteoblastic genes

**Figure 4:**
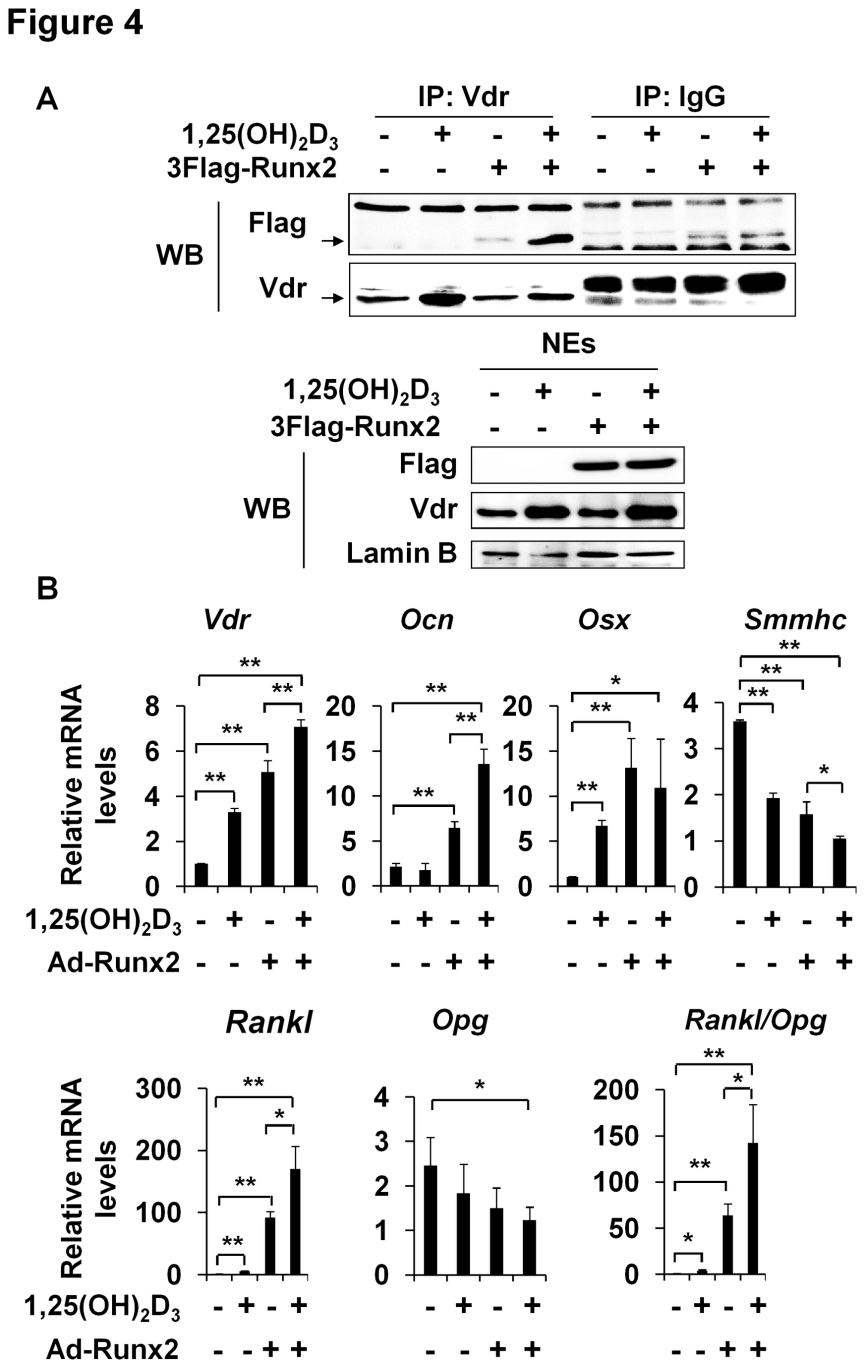
Physical interaction between Vdr and Runx2 and the expressions of bone related genes. (A) HEK293 cells were transfected with Flag-Runx2 and treated with 1,25(OH)_2_D_3_ (1 x 10^-7^ mol/l) for 24 hours. Nuclear extracts (NEs) were immunoprecipitated with anti-Vdr antibody and then immunoblotted with anti-Flag or anti-Vdr antibodies (upper panel). NEs were also immunoblotted with anti-Flag, anti-Vdr, and anti-Lamin B antibodies (lower panel). (B) Rat VSMCs were infected with Ad-LacZ or Ad-Runx2 vectors (at 50 moi) for 4 hours and then treated with or without 1,25(OH)_2_D_3_ (1 x 10^-7^ mol/l) for 42 hours. Relative mRNA levels of *Vdr*, *Ocn*, *Osx*, Rankl, *Opg*, and Smmhc were determined by real-time RT-PCR. Data are the means ± SDs of three separate experiments. Statistical analysis was analyzed using the unpaired Student’s *t*-test. *P<0.05 ; **P<0.01.

Vdr is known to bind to Runx2 in osteoblasts [25], and together these two factors activate osteogenic target genes [25,26]. To determine whether Vdr directly binds Runx2, immunoprecipitation was performed using HEK293 cells. HEK293 cells, transfected with or without 3Flag-Runx2, were treated with 1,25(OH)_2_D_3,_ immunoprecipitated with anti-Vdr antibody, and probed with anti-Flag antibody. As shown in Figure 4A, Runx2 co-precipitated with Vdr only in 1,25(OH)_2_D_3_-treated-Runx2-transfected cells. Control immunoprecipitation of normal IgG showed no increase in Runx2 by 1,25(OH)_2_D_3_ treatment. These results suggest that Vdr interacts with Runx2. 

1,25(OH)_2_D_3_ increased the mRNA levels of *Vdr, Osx*, and *Rankl*, but decreased *Smmhc* mRNA levels, whereas Runx2 overexpression increased the mRNA levels of *Vdr*, *Ocn*, *Osx*, and *Rankl*, but decreased *Smmhc* mRNA levels (Figure 4B). Furthermore, Runx2 plus 1,25(OH)_2_D_3_ additively increased the expressions of the mRNAs of *Vdr*, *Ocn*, and *Rankl*, but decreased those of *Opg* and *Smmhc*, and thus, sharply increased the *Rankl/Opg* ratio (Figure 4B). *Ocn* and *Opg* mRNA levels were not changed by 1,25(OH)_2_D_3,_ and no additive effects were observed in the expression of *Osx* (Figure 4B). Taken together, these results indicate that the interaction between Vdr and Runx2 additively induces the expressions of osteoblastic genes. 

### Effects of vitamin D_3_ on osteoblastic differentiation and calcium deposition in mice

**Figure 5:**
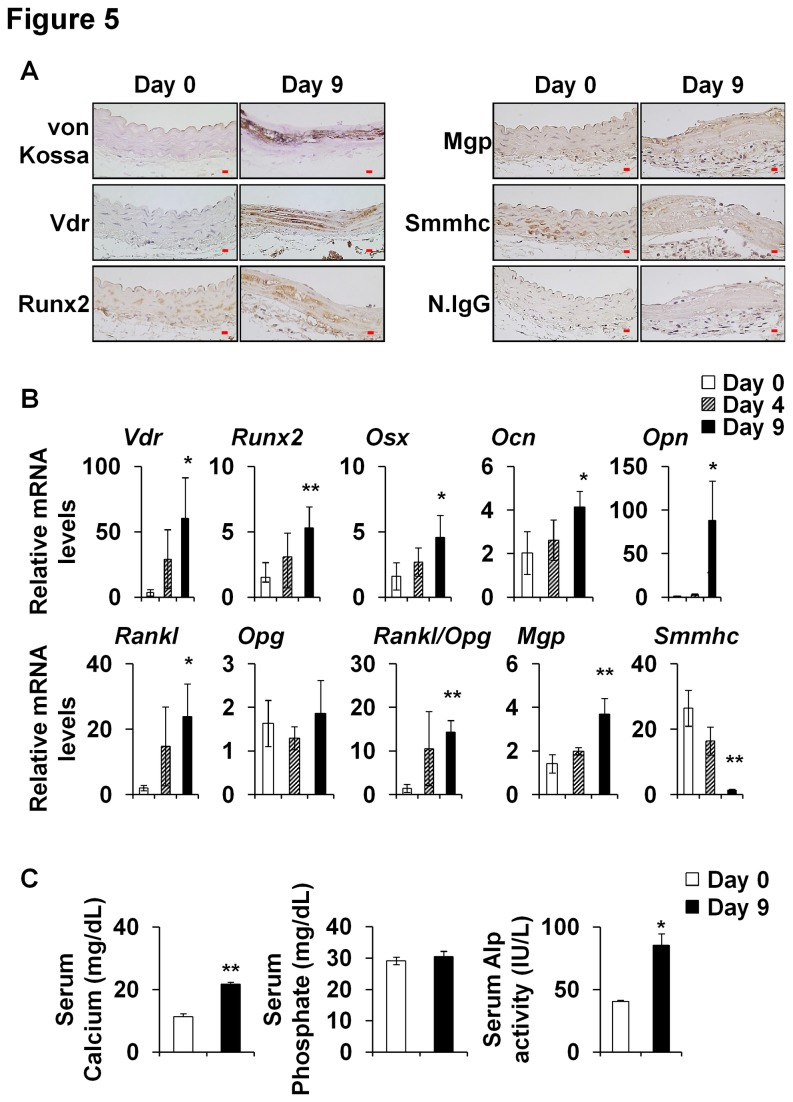
Vitamin D_3_-induced VC and the expression of bone related genes in the aortas of wild type mice. After administering high-dose vitamin D_3_ (6 x 10^5^ IU/kg of body weight) injection to wild type mice as described in Materials and Methods, aortas were obtained at day 0 (baseline), day 4 and day 9 and subjected to immunohistochemical or real-time RT-PCR assays. (A) Calcium deposition was observed by von Kossa staining. Vdr, Runx2, and Mgp protein levels were increased but Smmhc was decreased in calcified regions. Original magnification, X400 (Scale bar=10 μm). Mice (n=6-8) per group. Normal IgG (N.Igg) was used as an internal control. (B) The mRNA levels of *Vdr*, Runx2, *Osx*, *Ocn*, *Opn*, Rankl, *Opg*, *Mgp*, and Smmhc were measured by real-time RT-PCR. Data are group means ± SD (n=6-8/group). (C) Serum calcium and phosphate levels, and Alp activities were measured. Data are group means ± SD of experimental groups (n=6-8). Statistical analysis was performed using the unpaired Student’s *t*-test. *P<0.05; **P<0.01.

VC was induced in mice by administering vitamin D_3_, as previously described in the rat [40]. Injection of vitamin D_3_ subcutaneously for 3 consecutive days triggered VC in the aortic medial layer (Figure 5A). In addition, mineral deposition was observed in kidney and lung (Figure S1), but not in other tissues, such as, brain, liver, stomach, pancreas, spleen, and small intestine (data not shown). The relative mRNA levels of *Vdr*, *Runx2*, *Osx*, *Ocn*, *Opn*, *Rankl*, and *Mgp* in aortas were also gradually increased by vitamin D_3_ administration (Figure 5B), whereas the mRNA levels of *Smmhc*, a smooth muscle cell marker, gradually diminished (Figure 5B). Serum calcium levels and Alp activities were increased in vitamin D_3_ treated mice, but with no change in serum phosphate concentration was observed (Figure 5C). These results indicate that vitamin D_3_-mediated VC was associated with the upregulations of bone-related genes in calcified sites in our mouse model.

### Protection from VC induced by vitamin D_3_ in Vdr^-/-^ mice

**Figure 6:**
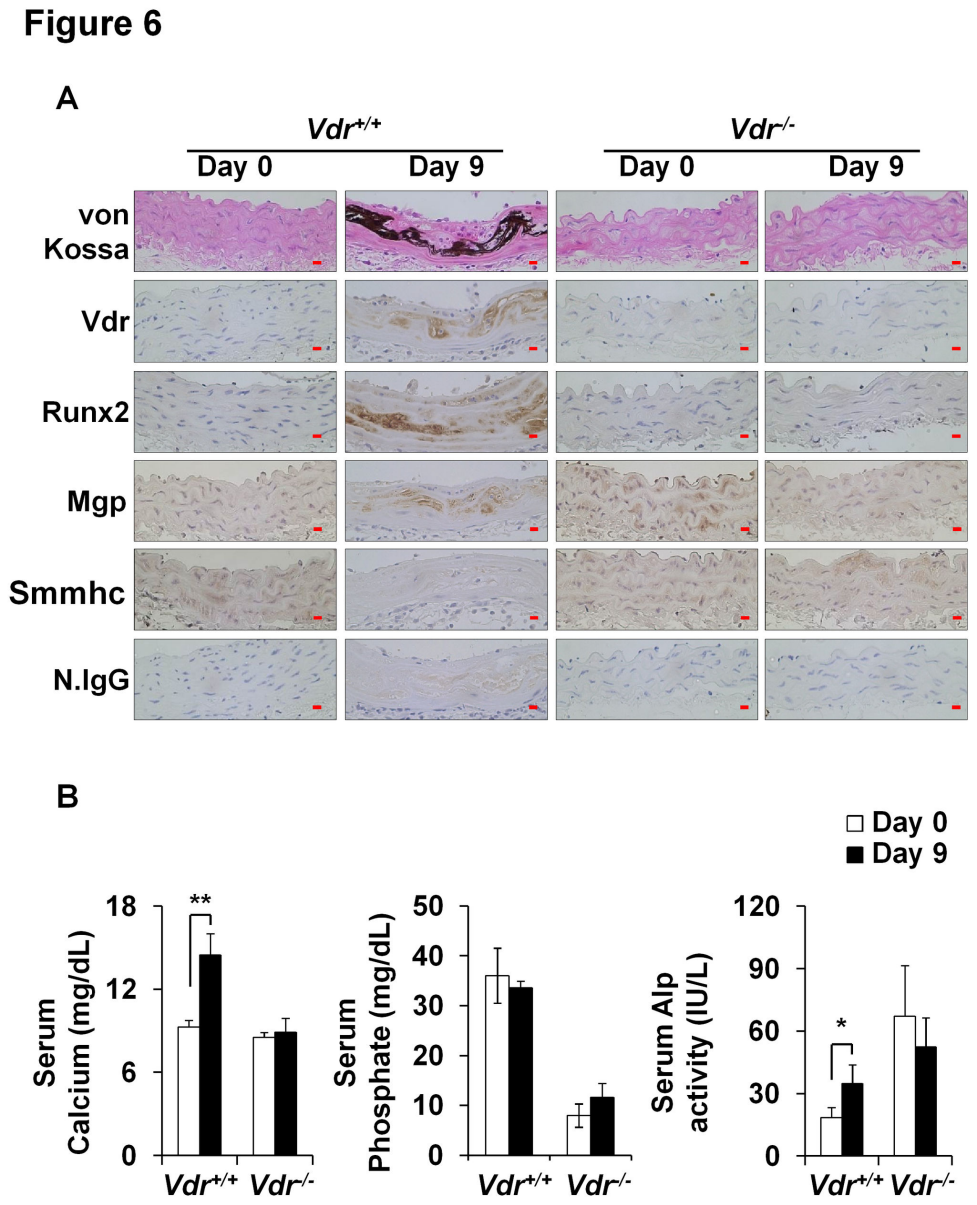
Protection from vitamin D_3_-induced VC in Vdr^-/-^ mice. Sixteen-week-old wild type (Vdr^+/+^) and Vdr^-/-^ mice were injected with vitamin D_3_ (8 x 10^5^ IU/kg of body weight). (A) Regions of calcification were detected by von Kossa and immunohistochemical staining. Vdr, Runx2, and Mgp protein levels were elevated in calcified regions in Vdr^+/+^ but not in Vdr^-/-^ mice. Original magnification, X400 (Scale bar=10 μm). Control, n=5; vitamin D_3_, n=5. N.IgG was used as an internal control. (B) Serum calcium, phosphate, and Alp levels were also analyzed. Data are group means ± SDs (n=5/group). Statistical analysis was performed using the unpaired Student’s *t*-test. *P<0.05; **P<0.01.

We also investigated vitamin D_3_-mediated VC in Vdr^-/-^ mice, which have similar calcium, lower phosphate, and higher Alp serum levels than wild-type mice [33]. Unlike their wild type littermates (*Vdr* ^*+/+*^), mineral deposition was not observed in the aortas of *Vdr* ^*-/-*^ mice (Figure 6A). Furthermore, high-dose vitamin D_3_ did not increase the protein expressions of Vdr, Runx2, and Mgp in *Vdr* ^*-/-*^ mice as it did in WT mice (Figure 6A). In addition, high-dose vitamin D_3_ did not increase serum calcium or Alp activity in *Vdr* ^*-/-*^ mice (Figure 6B). These results indicate that *Vdr* deficiency protected mice from the aortic deposition of mineral induced by high-dose vitamin D_3_.

### Protection of VC induced by vitamin D_3_ in *Runx2* ^*+/ΔC*^ mice

**Figure 7:**
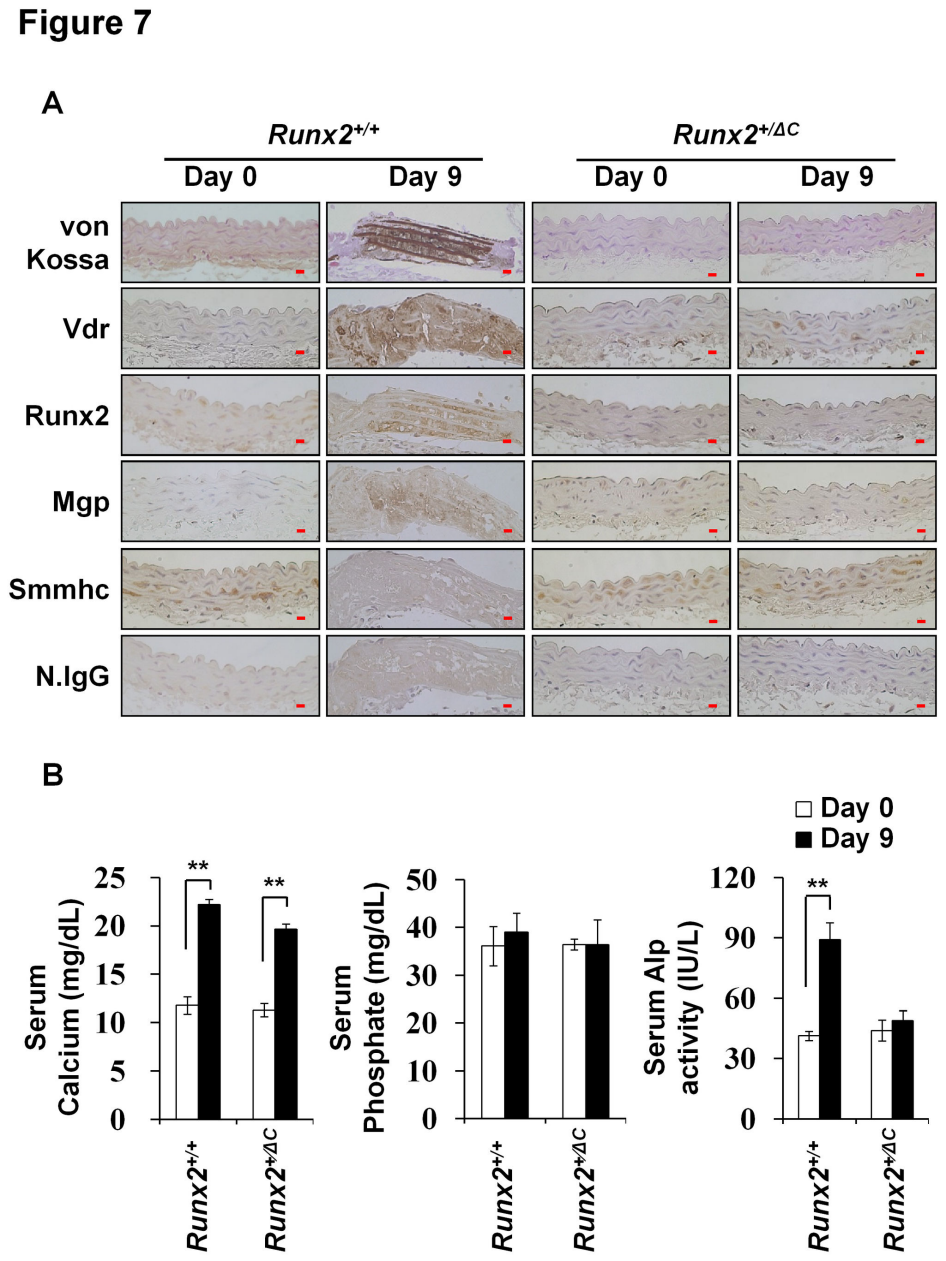
Protection from vitamin D_3_-induced VC in *Runx2* ^+/ΔC^ mice. Eight-week-old Runx2^+/+^ and *Runx2* ^+/ΔC^ mice were injected with vitamin D_3_ (6 x 10^5^ IU/kg of body weight). (A) Calcified regions were detected by von Kossa staining. The protein levels of Vdr, Runx2, Mgp, and Smmhc in these regions were assessed by immunohistochemical staining. Original magnification, X400 (Scale bar=10 μm). Experimental groups (n=5-8). N.IgG was used as an internal control. (B) Serum calcium, phosphate, and Alp levels were also measured. Data are group means ± SDs (n=5-8/group). Statistical analysis was analyzed using the unpaired Student’s *t*-test. *P<0.05; **P<0.01.

Since Runx2 expression is abrogated in *Vdr* null mice, we examined whether high-dose vitamin D_3_-induced VC was blunted in *Runx2* ^+/ΔC^ mice. To test this hypothesis, *Runx2* ^+/ΔC^ mice were injected with vitamin D_3_ and matrix mineral deposition and the protein levels of Vdr, Runx2, Mgp, and Smmhc in aortic tissues were assessed by von Kossa staining and immunohistochemical analyses, respectively. Vdr and other bone related markers (Runx2 and Mgp) were not induced by vitamin D_3_ in *Runx2* ^+/ΔC^ mice (Figure 7A). Furthermore, as was observed in Vdr^-/-^ mice, vitamin D_3_-inudced osteoblastic factors and mineral deposition were inhibited *Runx2* ^+/ΔC^ mice, despite increased serum calcium levels (Figures 7A and B). On the other hand, phosphate and Alp levels were unchanged by vitamin D_3_ in *Runx2* ^+/ΔC^ mice (Figure 7B). The protection from VC observed in *Runx2* ^*+/ΔC*^ mice in the presence of elevated levels of calcium suggests that VC is not caused by passive precipitation and that it requires active participation of Runx2 and Vdr. 

## Discussion

The results of this suggest functional cooperation between Vdr and Runx2 in the upregulations of their expressions, osteogenic gene expression, and mineral deposition are causally associated with VC. Furthermore, immunoprecipitation revealed that Vdr and Runx2 physically interact with each other. In addition, functional cooperation between Vdr and Runx2 with respect to their expressions and the expressions of osteogenic factors were found to play a key role in the procalcific effects of vitamin D_3_. Finally, our *in vitro* findings were supported by observation made *in vivo* our high-dose vitamin D_3_-induced VC mouse model. 

The expressions of Vdr and Runx2 additively increased the mRNA expressions of downstream osteogenic genes, and selective siRNA knockdown of *Vdr* and *Runx2* reciprocally inhibited the protein/mRNA expressions of Vdr and Runx2 in VSMCs. Indeed, vitamin D_3_-induced VC and the upregulations of bone related genes were completely diminished in *Vdr* ^*-/-*^ and in *Runx2* ^*+/ΔC*^ mice.

In osteoblasts, it has been shown that Vdr suppresses Runx2 expression by binding to VDRE in Runx2 promoter and that Runx2 autonomously suppresses its own expression [45]. However, the osteoblast-specific deletion of *Vdr* did not influence Runx2 expression in bone [46]. In the present study, 1,25(OH)_2_D_3_ increased Runx2 expression in VSMCs and *Vdr* played an essential role in vitamin D_3_-induced VC *in vivo*. These findings indicate a cell type specificity of Runx2 expression in response to vitamin D_3_ treatment. Further studies are needed to dissect the mechanisms responsible for the differential regulation of the *Runx2* gene in VSMCs and osteoblasts. 

We used a high concentration of 1,25(OH)_2_D_3_ (10^-7^ mol/l) to determine the molecular mechanism responsible for the transdifferentiation of VSMCs into osteoblast-like cells in cell culture systems because the expressions of Vdr, Runx2, and Smmhc were prominent and comparable to those of *in vivo* phenotypes at this concentration. In the present study, combined treatment of 1,25(OH)_2_D_3_ and Runx2 additively increased the expressions of *Vdr*, *Ocn*, and *Rankl*. *Ocn* is a skeleton-specific non-collagenous protein and was found recently to act as novel hormone signaling insulin secretion in the pancreas and testosterone synthesis in testes [47,48]. In the present study, *Ocn* expression was increased in VSMCs by Runx2 and further upregulated by Runx2 and Vdr. The significance of Ocn in the transdifferentiation of VSMCs is unclear, and it needs to be determined whether Ocn is involved in vitamin D_3_-mediated VC. In the present study, we also observed upregulattion of the Rankl/Opg pathway. More specifically, 1,25(OH)_2_D_3_ and Runx2 additively increased *Rankl* and decreased *Opg* levels, and thus, sharply increased the *Rankl/Opg* ratio. Reactive oxygen species and inflammation-mediated increases in Rankl have been previously reported to be important triggers of VC [4]. Furthermore, it has also been reported that Runx2 increases Rankl and contributes to VC by regulating Bmp2 and bone-related proteins, such as, Mgp [49,50]. These findings suggest that modulation of the Rankl/Opg pathway by Vdr and Runx2 plays an important role in the development of high-dose vitamin D_3_- induced VC. 

It has been established that VC development shows a U-shaped tendency with respect to serum vitamin D_3_ levels [29,30]. Recently, *Vdr* deficient mice fed a semi-synthetic western diet containing 2% calcium and 1.25% phosphate and *LDL receptor* deficient mice fed a semi-synthetic diet containing low levels of vitamin D_3_ were found to show aortic calcification and upregulations of osteogenic factors [30] and of low-dose Vdr activators that protect against VC [51]. In the present study, VC was clearly induced in mice by high-dose vitamin D_3_ injection as has previously been reported in rat models [40]. Furthermore, *Vdr* ^*-/-*^ and *Runx2* ^*+/ΔC*^ mice show strong inhibitory effects on high-dose vitamin D_3_-induced VC, and in these mice, osteogenic markers did not increase in blood vessels. In a previous murine study, smooth muscle cell-specific Runx2 deletion was found to inhibit high fat diet induced VC [52]. In the present study, despite increased serum calcium or Alp levels, the protective effects on VC in Vdr^-/-^ and *Runx2* ^*+/ΔC*^ mice indicate that both Vdr and Runx2 act independently of these factors, which suggests that vitamin D daily allowances be strictly adhered to particularly CKD patients [32]. Alp activity was not changed by vitamin D_3_ in neither Vdr^-/-^ or *Runx2* ^*+/ΔC*^ mice, but an increase was observed in WT mice. Interestingly, basal levels of Alp activity were high in Vdr^-/-^ mice, but similar in *Runx2* ^*+/ΔC*^ mice and WT mice. Vdr might be a negative regulator of Alp activity in bone tissues [46]. Although serum Alp activity upregulation by vitamin D_3_ reflects VC quite well, further studies are required to clarify the contribution made by Alp activity to the induction of VC.

Collectively, our findings show regulations of *Vdr* and *Runx2* at the gene level and functional interaction between Vdr and Runx2 are required for the regulations of VC-related osteogenic genes and mineral deposition in response to vitamin D_3_ in VSMCs. In particular, a deficiency in *Vdr* or *Runx2* completely abrogated vitamin D_3_ responses in our *in vivo* models. This study presents new evidence that targeting the expression levels of *Vdr* and *Runx2* and/or of the functional interaction between Vdr and Runx2 in vascular tissues provides a potential means of reducing cardiovascular morbidity and mortality caused by VC.

## Supporting Information

Figure S1 **High-dose vitamin D_3_-induced kidney and lung calcification.** (A) In kidney, mineral deposition was visualized by Alizarin red S or von Kossa staining at the walls of arcuate arteries, interlobular arteries (Arrow), and periglomerular arterioles in the cortex. Arrow head: Glomerulus. (B) In lung, mineral deposition was observed around alveolar. A: Alveolus; B: Bronchus. Magnification X1 and X400 (Scale bar=50 μm).
(TIF)
