# Supplementary figures and images for: Functional Cooperation between Vitamin D Receptor and Runx2 in Vitamin D-Induced Vascular Calcification
Source: PLoS One. 2013 Dec 12;8(12):e83584. doi: 10.1371/journal.pone.0083584 (PMC3861528; doi:10.1371/journal.pone.0083584)

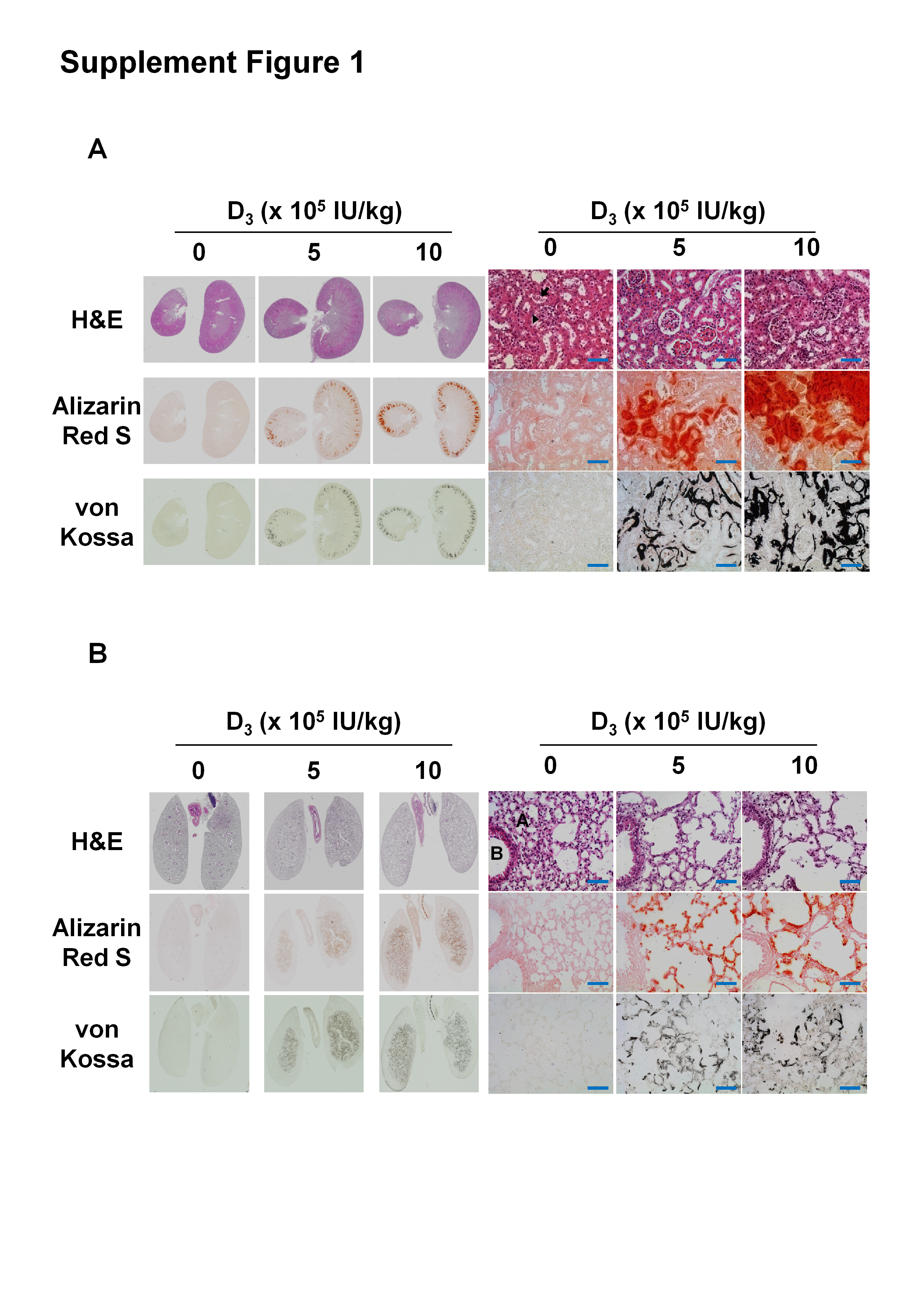

Supplement: Figure S1 — High-dose vitamin D3-induced kidney and lung calcification. (A) In kidney, mineral deposition was visualized by Alizarin red S or von Kossa staining at the walls of arcuate arteries, interlobular arteries (Arrow), and periglomerular arterioles in the cortex. Arrow head: Glomerulus. (B) In lung, mineral deposition was observed around alveolar. A: Alveolus; B: Bronchus. Magnification X1 and X400 (Scale bar=50 μm). (TIF) [file pone.0083584.s001.tif]
